# Erratum: The Botanical Drug PBI-05204, a Supercritical CO_2_ Extract of Nerium Oleander, is Synergistic With Radiotherapy in Models of Human Glioblastoma

**DOI:** 10.3389/fphar.2022.937601

**Published:** 2022-05-30

**Authors:** Frontiers Production Office

**Affiliations:** Frontiers Media SA, Lausanne, Switzerland

**Keywords:** glioblastoma, radiotherapy, PBI-05204, apoptosis, oleandrin, DNA repair

Due to a production error, an author name was incorrectly spelled as “Stefano Martelluci”. The correct spelling is “Stefano Martellucci”.

The publisher apologizes for this mistake. The original version of this article has been updated.

